# Therapeutic Notes

**Published:** 1907-07-20

**Authors:** 


### Therapeutic Notes.

### The Uncertain Action of Strophanthus.

The difficulty of obtaining a drug in a pure state has been the cause of the discontinuance of the use of more than one valuable medicinal product, but in few instances does the quality of a drug vary to such an extent as in the case of strophanthus seeds. While the quality of many drugs can be gauged by their outward appearance, and the presence of foreign matter detected almost at a glance by experienced druggists, it is extremely difficult—in fact, almost impossible—to distinguish the different varieties of strophanthus without applying chemical tests to each seed. For very many years attempts have been made to obtain a free supply of the genuine drug, and although it is now possible to procure the seeds of *S. Kombé* in a pure state, seeds which are not genuine are frequently offered on the market.

In 1886 E. M. Holmes described six varieties of seeds which had found their way into commerce (*Pharmaceutical Journal* [3] 16, 778); and in 1888 six varieties were also found in French commerce, the only species giving the same reaction with sulphuric acid as the Kombé being the seeds of *Strophanthus hispidus*. The risk entailed by the use of seeds widely varying in activity is obviously very great; but it is satisfactory to know that at the present time the genuine unmixed drug can be obtained from reliable firms, who are naturally obliged to charge higher prices than those at which mixed seeds are obtainable. F. M. Moir, the secretary of the African Lakes Corporation, Limited, attributed the difficulty in obtaining unmixed pods to the fact that the plant grows at considerable distance from European habitations, and that the natives sent to collect the pods would bring other pods similar in size which could not be distinguished from them unless the plants were seen growing (*Pharmaceutical Journal* [4] 22, 312). Although every endeavour was made by the African Lakes Corporation to secure the collection of genuine strophanthus only, it was not until 1903 that the pods submitted by the Corporation to experts were found to consist entirely of genuine Kombé. In 1906 the exclusive handling of *Strophanthus Kombé* was transferred by the African Lakes Corporation to Messrs. Oppenheimer, Son and Co., an arrangement having been made that the seeds exported to Great Britain should be collected under the direction of botanists.

### Salicylic Acid Derivatives.

That large doses of salicylic acid or its congeners must be given in acute rheumatism is now generally recognised. Clarke (" Amer. Jour. Med. Sci." September, 1906) gives 10 to 20 grains every hour till toxic symptoms appear, as they do on an average after 200 grains have been given. The drug is then stopped, and not again prescribed till all the toxic symptoms have disappeared. Children take salicylates well. Longmead (" Lancet," June 30th, 1906) found as the result of careful observation, that the toxic dose varied as much as in adults, but notes that 100 to 300 grains may be given per diem to children of six to ten years of age. Occasionally symptoms of acid poisoning occur, and acetone may be recognised in the breath and urine. Large doses of bicarbonate of sodium should then be given (one dram every hour). A large number of esters of salicylic acid have been introduced, some of which, such as acetyl-salicylic ester, are much used. Among the newer ones may be mentioned—

*Benzosalin.*—This is a compound of benzoin and salicylic acids. The dose is 15 grains three or four times a day in subacute cases, but more might be given when the toxic symptoms are severe. Langheld ("Therapist," December 15th, 1906) states that it is not broken up in the stomach, and consequently does not interfere with digestion, and that it has no injurious effects on the heart or kidneys. He considers it valuable in cases in which the ordinary sodium salt is not well tolerated. It is prepared by Hoffmann, La Roche and Co., Baden.

*Salimenthol* is an ester of salicylic acid with menthol. It is a fluid substance almost devoid of taste and smell, and miscible with all ordinary solvents. Internally it may be given in drops, the dose being 15 to 30 thrice daily, or oftener, according to the case. Capsules are also sold, which afford a convenient method of administration. The drug may also be applied externally, a method of treatment which has been somewhat largely adopted with the various salicylate preparations of late years. For application either " Samol " may be employed, which is sold in tubes, and consists of 15 per cent. of salimenthol in an ointment basis, or an ointment may be prescribed with lanoline or vaseline of a similar strength. In subacute cases and in cases of muscular rheumatism a 4 per cent. solution in rectified spirit of wine may be used as a liniment, or an oily preparation may be prescribed, 12 per cent. salimenthol in a mixture of equal parts of chloroform and oleum hyoscyami. Salimenthol is not miscible with water, dilute alcohol, or glycerine. It has been found useful in rheumatic and other painful conditions of the muscles and nerves, the menthol apparently giving it a wider application as an analgesic than is usually possible with the salicylic preparations.

*Salen* is intended for external application only, like the methoxyl methyl ester. It is a mixture of the methyl and ethyl glycolic acid esters of salicylic acid, and is a colourless liquid at ordinary temperatures, though the bodies of which it is composed are crystalline solids. It is soluble in the usual media—equal parts of olive oil and castor oil, or of olive oil and chloroform are recommended. If one of these solvents be adopted, the salen should be added up to 50 per cent., and the affected joint should be rubbed with a drachm or so thrice daily, and in the intervals be kept covered with wool. As an alternative, the pure substance may be applied as a paint. The advantages of salen are that it is practically free from odour, which is such an obstacle to the employment of oleum gaultheriæ, and it does not, like some salicylic esters, irritate the skin and lead to the occurrence of eczema. Salen is prepared by the Society for Chemical Industries in Basle.

